# The Experimental School in Bonneuil-sur-Marne…with Commentary from a North American Context

**DOI:** 10.3389/fpsyg.2017.01794

**Published:** 2017-10-20

**Authors:** Catherine Vanier, Kareen Malone

**Affiliations:** ^1^Espace Analytique, Paris, France; ^2^Department of Psychology, University of West Georgia, Carrollton, GA, United States; ^3^Après Coup Psychoanalytic Association, New York, NY, United States

**Keywords:** Mannoni, psychoanalysis with children, anti-psychiatry, mental health hospitals, Lacanian psychoanalysis

The Experimental School in Bonneuil-sur-Marne was founded by Maud Mannoni in 1969 as an institution for children and adolescents with difficulties. Mannoni wanted Bonneuil to be a different place, a living space rather than a space of care. “At Bonneuil,” Mannoni (1976) would say” we receive children troubled by the system—whether it is the education system, the family or the social system. And the adults who take care of them, such as myself, could also be defined as troubled by the system, in the sense that they cannot bear working as caregivers in hospitals or as teachers in secondary schools” (p. 21).

Initially, about 20 (now 40) children, all of then outside the traditional education system and who had been labeled as mentally retarded, psychotic, autistic or suffering from severe behavioral problems, were received at the School during regular school hours, from 9 a.m. to 4 p.m. in a building located in the suburbs of Paris^1^. Together with adults, they helped each other take care of this living space—shopping, cooking, cleaning—and set up activities in which both adults and children could choose to participate: school work, painting, sculpture, music, theater and others, depending on their own suggestions for such activities. Some children can also stay the night or over the weekend in different facilities nearby; others return to their families at the end of each day. Mannoni says that at the beginning, the children would ask: “Are we a school for crazy people?” But by 2 months after the School had opened, such questions might receive the following answer: “Who here needs a crazy person to feel good?” (Mannoni, 1970, p. 204).

The founding ideas for Bonneuil were born from an encounter between psychoanalysis and the anti-psychiatry movement of that time (e.g., R.D. Laing, David Cooper, see Boyers, 1971). In all the texts in which Mannoni speaks about Bonneuil, she obviously refers to Lacan, but also to Winnicott, who had been her supervisor. She incorporated Winnicott's idea that psychosis was not simply a pathological process, but also and especially a reaction against an extremely conflictual life situation. These children therefore needed a place where they could live in another way.

From the beginning, Mannoni wanted a place adhering to the principles of anti-psychiatry and opposed to the hospitals of the time, which indeed were most often places of confinement and segregation. Instead, she founded a school able to accommodate madness, while avoiding labeling children or making them into “objects” of care. The adults who joined forces with her were young psychoanalysts, psychiatrists, and educators.

These adult “staff” all worked and today still continues to work differently than many current approaches. They do not refer to expert knowledge, which, as Mannoni believed, would only serve to deepen the staff's own defenses toward the child's subjective conflicts and difficulties and obscure the effect of truth inherent in the child's “symptomatic discourse”. In accord with Mannoni's vision, we thus continue to receive and welcome children with all of their difference and anxiety, without categorizing them or filling out forms. And through different activities we try to offer them the possibility of doing something *with* their symptom, in order to make their life more bearable, and help them discover that they are not simply the sick children so often marginalized and objects of treatment, but also subjects who can take the risk of confronting their desire.

There is no psychoanalytic treatment for the children at Bonneuil, no interpretation, but instead our work is framed by a psychoanalytic reading of what is going on at the School. If, at a particular moment in his journey, a child wants to speak to an analyst, he is sent outside the School, to someone who does not work at the institution. Bonneuil is a transitional place, where children and adults travel a part of their journey together. We do not focus on a child's diagnosis of mental illness, but offer to do things with the child, which helps him discover what he would like to do and who he could become. At Bonneuil, the adults, children with difficulties and so-called normal children all participate in these activities together. So much so that, as Mannoni would say, you can no longer tell who is mad and who isn't. While visiting the school when their son was admitted, one couple of parents told me: “We were really surprised to see everyone working on something. We were trying to figure out who were the patients and who the caregivers, but we couldn't really tell.”

Some children are schooled at Bonneuil. They can prepare for their exams and on leaving us they go back to the state education system; others turn toward learning a trade; others still join communities outside the Paris region, where they have been able to make a place for themselves. Sometimes very little is needed for this kind of progress to happen: receiving the children, listening, keeping an attitude of openness toward other spaces.

This openness toward the outside is what Maud Mannoni called *institution éclatée*, which we could perhaps translate as an *inside-outside institution*. She came up with the idea of an institutional framework which, while constituting a permanent basis, would allow for gaps and openings, for projects directed toward the outside. Mannoni found it important to allow for the possibility of an elsewhere, such as for example going to work with a craftsman or spending a period of time outside Bonneuil, with foster families in the countryside. While remaining a place of refuge, Bonneuil can suggest that certain essential things also happen elsewhere. Through this oscillation between one place and another, the subject can emerge and question his desire. Maud Mannoni spoke about the inside-outside institution as a version of the *Fort-Da* game (Freud, 1955/1920), an oscillation between the here and there. Every time the child leaves for work or spends some time away, she would say: “Through these comings and goings, a signifying space opens up, in which the child is set to be lost, in order to give himself the illusion of being reborn. Contrary to the *Fort-Da* game, the mother does not leave, but the child is put in a situation where he has to leave her and leave Bonneuil. If this separation is successful (which cannot happen at just any moment), the child becomes a subject, insofar as he is absent as an object” (Mannoni, 1973, p. 74). She often emphasized this existence of an elsewhere, which is also a possibility for the child to reject – to “vomit,” as she put it—the institution. “It is advisable that a child changes place as soon as he settles into an adaptive stereotype. The break between the two places gives rise to the healing effects of change” (Mannoni, 1973, p. 81). A different place of work, a stay in the countryside, going to the theater, to museums and concerts, with different adults—all these become the child's opportunities to discover, in the game of presence-absence, who he is and who he would like to be. Being an “experimental school” does not mean that at Bonneuil children are the subjects of an experiment, but that it is not simply a place where traditional rules are applied: it is a place which leaves room for inventiveness and the unexpected.

In spite of Mannoni's radical non-conformism, in 1975 Bonneuil was officially recognized as a day hospital. However, the struggle that she was forced to wage at the time—because, as she emphasized, “these kinds of marginal operations will necessarily upset the administrative structures governing us”—is still alive in 2017. Today more than ever before, our increasingly more regulated and security-obsessed society imposes various operating rules, not always compatible with the spirit of our institution. The threat hanging over Bonneuil is therefore still very present and the fight is far from over.

In 1975, there was a film made at Bonneuil (Séligmann, 1974) and it shows a glimpse of the work that we have been and are doing there. The following conveys some of these concrete activities which demonstrate the way in which what we do indicate Mannoni's philosophy of an inside/outside school as a way to frame certain subjective conditions that allow participants a different relation to their desire. Providing the opportunity for children to work externally is again part of the idea of an inside-outside institution. Against the background of permanence, i.e., the school itself, the openings toward the outside, such as field trips, involvement in outside workplaces and apprenticeships, are also created within a specific alternation; what is at stake is the oscillation between a project and a flight; the movement prevents the death that threatens any institution closed upon itself.

Within this “institutional” practice, children can participate in the real working life of adults, and they come back transformed by the experience they have acquired in this back-and-forth movement, between one place and another. Some children, who did not want to stay at Bonneuil, have given meaning to their lives by working in a restaurant, a bakery or a mechanic's workshop. In terms of learning, Mannoni (1976) would say that, “we cannot compare the situation of a child going to work with a carpenter and has the experience of creating something with adults, and that of a child receiving a carpentry lesson from a teacher at a technical college. The child situates the vocational institution as a dead place. But working in the craftsmen's workshop is a discovery of desire through chaos—a desire that can also become a wish to return to a more ‘classical’ form of study” (p. 60).

Bonneuil offers children the possibility of working outside the School, but it is also a school in itself; our team includes teachers from the state education system. The classes are divided into two large groups: the “Communal” school is for the smaller children, the “Fac Spé” for the older ones. In terms of schooling, it is the non-coincidence with the official pedagogical discourse that has been effective in its effects with the children. On the one hand, we have maintained a connection with the normal reality of school education (courses and diplomas); on the other hand, the structure of the School has remained open toward the outside (a journal, videos, excursions). Moreover, there is a continuous questioning of educational ideology through discussion groups involving children and adults from the school. Replacing competition with mutual aid has also been an essential part of this framework. As Alain Vanier explains, “we could say that anti-pedagogy, like antipsychiatry, is not a pure negation of psychiatry or pedagogy, but an attitude allowing to uphold the questioning of the adult's and child's desire.” (Vanier and Richer, 1976) To make this possible, we must constantly be listening to the discourse that is produced in our discussions so that the stakes of desire are as clearly heard as the information that is the usual and often total focus of education. If the children wish to do so, they can sit the traditional exams of state education – the Certificate of National Education, the Baccalaureate—accompanied by the adults they themselves choose, who sometimes make them work in groups, sometimes by themselves.

Perhaps I can also convey something of everyday life at Bonneuil. Each day begins with *la causette—*“a time to talk,” which brings children and adults together. On the blackboard, we put the different activities offered for the day, with the names of those wishing to participate. This is also the time to talk about what is going on in the children's lives or, perhaps, an occasion to throw a fit about it. Once we have together agreed on the daily menu, some of us go shopping or gardening, or leave for work. Then we prepare lunch in the kitchen. The work of the adults who accompany the child throughout these activities allows the child to discover the reality of the world around him. For an autistic child, for example, this reality, which he tries to shield himself from at all costs, cannot be symbolized. Given the difficulty with symbolization, what he is doing is trying to protect himself from it. All that cannot be controlled becomes dangerous and can easily trigger panic. There are many consequences for this child who must protect himself without the shield of the symbol. Such a child is constantly trying to protect himself from and live without affect. The adult's presence and words give the child a possibility of experiencing reality and its affects without the threat of annihilation.

An important component of the time at Bonneuil is spent in the workshops. School activities take place in the morning, workshops most often in the afternoon. For Mannoni, these workshops were primarily meant to be places of encounter and, as she put it, of the transformation of the child's attitude under the effect of transference. They are not a pretext for therapy and are not supposed to lead to interpretations. Instead, the activity pursued in a workshop is a third element, a *doing* introduced between the adult and the child. The workshop is conceived of in relation to the outside world: at the end of the year we organize theater performances, exhibitions and concerts, which are open to the general public. This too is what makes us an inside-outside institution.

Inspired by Bonneuil, the practice of creating workshops and ateliers has now become commonplace in nearly all therapeutic spaces, but this poses a risk that they too will become part of the caretakers' arsenal, that they will become systematized and will lose their original subversive edge. Bonneuil cannot function as a model, but it may perhaps provoke a spark for invention in others. In the workshop, work must in fact be reinvented each time.

In our painting workshop, there is a ritual, at the beginning and at the end, when you can paint whatever you want on the large blank sheets covering the walls and the floor. There is only one rule: nobody can take the neighbor's place. A space limit and a time limit, with a beginning and an end: there are limits at Bonneuil and not doing harm to others is an absolutely essential one. Based on this limit, the children are free to draw whatever they want. Very quickly, they tend to abandon the stereotypical drawings they were used to and authorize themselves to draw in a genuine way.

Theatre workshops have produced a number of performances, and one performance, the children's production of *Alice in Wonderland* was staged at a Parisian theater. Here too, the aim is to move outside the walls of the School. Based on the story by Lewis Carroll and a voice-off narration of the storyline, the children invented their own lines and played roles they have chosen. Starting from this framework, they invent the play they wish to perform. Again, these are not deliberately therapeutic workshops; and only in this way, Mannoni would say, adopting one of Lacan's formulations, they may have a chance of actually becoming therapeutic, “as an additional benefit.”

There have been many controversies about psychoanalysis in its work with autistic^2^ or psychotic children. Many of these controversies have shed little light on the particularity and framing that actually goes into the work at Bonneuil. There are sometimes assumptions about an antagonism between psychoanalyst and the parents. In fact, one needs introduce a space for the desire of the child, which is different than “blaming the parents.” Most parents are at their wit's end and of course, carry their own suffering (Vanier, 2014). It is the child that is given priority.

Yet parents are involved. Many will prefer Bonneuil to any psychiatric hospital, especially if you think about such hospitals in the 1970's which were extremely rigid and closed places. At Bonneuil, every Monday morning was dedicated to a parents' meeting. These were run by Maud Mannoni herself. Parents would speak about what was going on in the home and the problems they were dealing with. At Bonneuil, we always give priority to the speech of the children and their parents. Today it is I who am in charge of these meetings, which are very important for the work we do with the children. It is indeed not always easy for the parents to tolerate the ways in which our inside-outside institution functions—the visits to the countryside, the openness toward the outside, which bring the children to detach themselves from their parents and lead their own lives. Mannoni (1976) said that she would never speak authoritatively in these meetings: “It is by identifying myself with the mothers,” she would say, “that I can make them more sensitive to the aspects of the struggle (which is larger than their child and themselves) and that I can make space for a discourse in which the parents can sometimes begin to support each other” (p. 189).

From the beginning until today, at Bonneuil we have always taken seriously what the parents have to say, without ever blaming them, just like we take seriously the discourse of the children who, through their symptoms, articulate something of their own truth. We take speech seriously, as the expression of the subject. This attitude has remained unchanged since the school was created. What has changed is of a different order.

In 1969, the mindset behind the creation of Bonneuil was anti-psychiatric, which for Mannoni meant a desire to avoid segregation—a segregation which she thought was here 2-fold: one was being excluded as a madman and again as a child. “Inside every one of us,” she wrote, “there is a place for the rejection of madness, that is to say, the rejection of our own repressed” (Mannoni, 1970, p. 243). Today, madness continues to be rejected, but it is no longer relayed through the psychiatric hospital. The rejection is of another kind: we try to annul it through a process of normalization and at all costs. The question is no longer of locking up children in psychiatric hospitals, but of integrating them, with all our might, in traditional schools, where they, most often fail because they cannot find a place for themselves. Later, when the school judges them incapable of following the standard course of learning, they are left at home, where they remain the charge of their distressed and exhausted parents. Today, no beds are being created, no places in hospital services to which children could be entrusted. Those families that can afford it end up looking abroad—in Belgium, Switzerland—for institutions that could receive them. There are no places left in France and creating new structures is economically impossible.

Anti-psychiatry is therefore no longer the answer—instead, the problem we are facing today is the process of mass “de-psychiatrization.” And because madness must always be “repressed,” our society excludes the mad in its own way: if they are adults, they are left on the streets or in prison; if they are children, they are left to their parents, as if madness did not exist. In the case of children, we are also no longer dealing with mental illness, and thus no longer need care institutions. The child is no longer psychotic (psychosis no longer exists) and instead joins the ranks of autistic individuals, who are now seen as disabled children in need of re-training. We are told that autism is a failure of learning in need of correction. We thus see a return to the normalization of orthopedics; the modern form of segregation has become a negation of the subject, at the expense of applying a veneer of standardized behavior attained by re-training or disciplining. These methods cannot but remind us of the nineteenth century educational methods of President Schreber's father. And yet, CBT treatment has already revealed its limits just as patients' rights associations have pointed out the many shortcomings of modern psychiatry. Children such as those we receive at Bonneuil need places to live that are different from the former institutions of traditional psychiatry, but still remain places of care.

It is therefore obvious why since its creation, Bonneuil has remained a thorn in the side of the authorities, and why this is still the case today. We do not ask the child to adapt to the society, but the society to adapt to the child, so that he can gradually tame the anxiety that cuts him off from the rest of the world and find a place that is his own. Bonneuil is a care facility because it remains a psychiatric day hospital for children: but it is a different kind of care, because we maintain the idea that the child's symptom must be heard as the manifestation of the subject. In Bonneuil, everything is organized so as to avoid negating this question through training and discipline, which would precisely try to eradicate the symptom. This is the basis of our work. At the same time, we are not trying to exclude pedagogy as a tool of adapting to the world; however, here too, it is not just *any* pedagogy and it is not given just *any* place.

Thus the struggle continues, and it is now our responsibility to keep this work alive, so that Bonneuil can continue to question—as it has always done, but in a way that reflects our time—the place of madness in today's society.

## Commentary from a north american context

### I. Why now?

There are a number of reasons that it is important to become aware of psychoanalytic work which counter reigning narratives of psychoanalysis among the disciplines of psychology, psychiatry, and with the educated public. The filming of the documentary, “The Wall,” now banned, but available in pirated versions, presented provocative juxtapositions between the words of analysts and scenes of a family with autistic children as well as highly edited interviews with psychoanalysts. These may well indicate problematic analytic formulations, but the overall effect was a mis-leading impression of outdated parent-blaming Lacanian analysts, making generalized proclamations about autism. The many questions about autism raised by the film are answered through its own polemic lens. For example, does the psychiatric diagnosis of autism, as it morphs into a spectrum, exactly parallel the meaning given by the interviewed analysts? But what is most striking is how divorced the implied attitudes within psychoanalysis, as formulated in relation to severely distressed children, seem to be given some truly innovative analytic work with psychotics and with autism or with many other distressed individuals—much of it partly inspired by Mannoni (see also O'Loughlin and Merchant, 2012).

Much of the skepticism regarding psychoanalysis and thus, by implication, of Mannoni's and Bonneuil's instantiation of it, has emerged from parents of autistic children. Forming their own advocacy groups, parents have sought to capitalize on biological causation as the explanation of their children's suffering and have looked to emphasize strengths and the status of autism not as a disease but as an matter of specialized educational approaches, a different neurological/cognitive style, so to speak, and on parallel with disabilities (Chamak, 2008; Laurent, 2012; Brownlow and O'Dell, 2013). Parental hostility to psychoanalysis has a number of sources, as noted by Vanier above, but also clearly entails a conflation of psychoanalysis with psychiatry. This mistaken conflation obviates any possible criticism of Bonniuel, even indirectly. Moreover, no one contests that the questions of education, social viability and recognition are vital. Recent patient groups, often at odds with their parents, have lobbied for more social recognition, contrasting themselves with neurotypicals. These new movements are strategies that speak to important issues, particularly social marginalization. Certainly, the École at Bonneuil is quite sensitive to this issue and the sources of the demands and activities of such groups. However, reducing humans to a disease category is one sort of misstep. Another form of marginalization entails the suppression of the irrationality and madness that characterize the human subject. This is a marginalization that identity politics cannot fully address (Berry, 2003; Chamak, 2008; Brownlow and O'Dell, 2013). Moreover, much of current criticism underestimates the different social contexts that impact deeply troubled children and their families, the professional traditions to address such children, and even the cultural context of diagnoses and of the diagnosed child/person's mode of integration into the social realm (Chamak and Bonniau, 2013)^3^.

More broadly, there is the continued dismissal of psychoanalysis and psychodynamic approaches in Anglo-phone countries, even in the face of the flawed experiments/data analysis promoting Cognitive and Behavioral approaches (Westen et al., 2004), and the alternative facts about the actual research results on therapeutic effectiveness of psychoanalytic inspired and oriented approaches (e.g. Shedler, 2010). Certainly, many ascribe to a CBT approach as collaborative, although the rhetoric seems contra-indicated as the patients seem totally subsumed under diagnostic categories, assessments, and protocols (see Shtayermman, 2016). Of course, the practices may differ. Nonetheless, the skewed view of data and theory as regards psychoanalysis has precipitated a professional crisis in psychology concerning clinical curriculum in the United States (Yakushko, in preparation). Yet in the case of Lacanian approaches, the seemingly immovable conviction that Lacan's reading of the psychoanalytic project must engender overly intellectual (Burgoyne and Sullivan, 1999), rigid, and conservative qua normative analysts (Malone, 2000; Van Haute, 2016) is so removed from its history of creativity, its attention to producing what is new, and its focus on desire. The Lacanian ethic of assuming responsibility for one's own singularity entails attending to that child or adult as a subject, not imposing one's theoretic grid upon him or her. This singularity is foregrounded even as it is theory of human subjectivity which crosses the boundary between the collective and the individual. So there is a long list of reasons for reading Vanier's story of the experimental school in Bonneuil-sur-Marne. It is not the Lacanism that many psychologists so predictably expect.

In its particular alliance with the anti-psychiatry movement of the sixties, Bonneuil suggests a different way in which one can counter the deadening diagnosis driven psychopharmacology that so permeates mental health discourse. Anti-psychiatry took humanistic forms in the United States or infused aspects of family therapy, as we see still in the work of Michael White (Freedman and Combs, 1996). It is true that Mannoni's version of anti-psychiatry and psychoanalysis differed from, for example, the approach of Felix Guarttari who was critical of what he saw as Mannoni's attention to familial dynamics (Dosse, 2011). This essay is not the place to argue out various forms of critical family theory nor re-visit the debates and evolution of anti-psychiatry. Clearly, patient advocacy groups suggest that generational dynamics are very important. These same groups suggest that the child's ‘connecting’ his or her desire to a place in the social link is also critical. Both of these dimensions are addressed at Bonneuil in a manner that echoes Mannoni's psychoanalytic approach. For Vanier and Bonneuil's founder, Maud Mannoni, it is psychoanalysis that offers a counter practice to the normalizing aims of the psy-disciplines and of many ghosts of the psychiatric institutions of the twentieth century. How we normalize today, as Vanier notes, may have changed.

Listening firstly to the voices of patients, children, and parents, Vanier's approach and psychoanalytic efforts to offer places and possibilities to those consigned to the low end of a faux pluralism of difference can never be static or prescribed. Both Vanier and Mannoni make explicit remarks that Lacanian dogma is as pernicious as other dogma to projects such as Bonneuil. This renders certain critiques of the psychiatry/psychoanalytic approach in France not directly relevant to the particular work at Bonneuil. Mannoni notes that it is a Lacanian misstep to confuse the functioning of the father's law with becoming institutional police; it is a dodge to fend off one's own discomfort with one's patients or one's own madness (Mannoni, 1999, p. 132). One's commitment to a psychoanalytic notion of the unconscious inscribes a domain of effects, but it does not prescribe norms of healthy functioning. In fact, we may say that Lacanian analysis is particularly marked by the obvious Freudian precept that whatever is normal and that not what whatever is deemed psychopathology are both variants on the theme of what it is to become human. This deeply held conviction, which is perfectly Freudian, informs how one is ethically oriented to the patient but also how one understands the person's relationship to the social (Vanier, 2004; Van Haute, 2016).

### II. Space and place

Maud Mannoni christened Bonneuil an institution éclatée, which Vanier translates as inside-outside institution. Now there are many meanings to this inside-outside. Vanier's translation is much nuanced. It refers to a way of providing possibilities for the children attending Bonneuil to enter into a new relationship to their own desire, and thus to their future. Bonneuil, unlike milieu therapy derived from Menninger (1939) does not work in a top down distribution of the expert knowledge from the psychoanalyst so that activities will be tailored to the “unconscious needs” patients (Freeman, 1960). Nor do the social components of Bonneuil model a “healthy” form of sociality which then complements individual analysis (Gralnick, 1989). The notion of inside-outside denotes a keen sense of how the spaces that the children occupy are demarcated and function. As the words “inside/outside” to describe an institution suggests, the spaces of Bonneuil are extremely important, as well as the movement between spaces. John Brenkman notes (Brenkman, 1999), that the spaces are often quite clearly marked. Personal analysis is outside the institution; it is personal space. The space for creative expression, like drawing, is “contained” by some rituals and rules, but then opens to all forms of what is drawn. Some drawings express terror and anxiety while others seem singular yet art that is available for others or a public. Adults may help secure this space, but they are not there as therapists. This may allow the art to become used by the child as a way toward self-expression and a “working through.” There are the outside places of work and travel. There is the schooling, which is kept separate from other undertakings, except in the overall experimental orientation. The child will not be oppressed in his style of learning, there is a critical awareness that pedagogy has its aims to mold knowledge and character; there is a questioning of one's desire, both of teacher and student as part of the process. That questioning is suppressed in the system of education but it allows a place of connection between what one desires and what one must and wants to learn (Vanier, 2004). It is this respect for the child—a respect that is grounded in the approach of Vanier (2014) in her private psychoanalytic work of children as well—that anchors the formal schooling at Bonneuil.

To work in another town, stay with another family, learn a trade “on site” means that the child moves back and forth between Bonneuil and other locations. This movement away from the institution, which by its nature may generate transference, and to another place introduces a gap. It is like the space between signifiers, wherein a difference can emerge. This difference can only be calibrated by the child who is in transition, and it opens the path of desire.

It seems that Lacan gave much attention to space as well as to the framing of that space in treatment. The mirror stage engages the dimension of space, indicating how the distance and closeness of others is psychologically intimate, constitutive, and perhaps fatal. The work on topology, which runs throughout Lacan, is again an engagement with space, indicating the necessary traversal of what “holds” the subject, implicating inside and outside in analytic work and in constitution of the subject of the unconscious. For example, one is never outside the transference, to comment “on” it. The metaphor as a practice of an institution of inside/outside is quite apt. Emergent as one's birth in the field of the Other (named, caught in generations of fantasy), the genesis of subjective desire in relation to the Other is a complicated affair in which need inmixes with the other's demand. The child navigates that subject whom he or she is supposed as being (see also Vanier, 2015). The delicate balance of this address to the subject from the Other, its reception, and dialectic, in-forms Bonneuil; it is a matter of words and acts, but not of interpretations nor of protocols. As in the psychoanalytic clinic, the work is driven by the child's timing and by the necessary recourse to invention rather than procedures and techniques.

Bonneuil does not eliminate symptoms. There is some truth in that symptom for the subject, which will be lost if the symptom is not allowed a place for expression and respected as what the subject has made as a response to his or her perceived situation (Vanier, 2014). It also involves a jouissance, a complicit enjoyment that makes it a bit harder to give up. If, as family therapists and Lacanians have often noted (Buccino, 2000), the child's symptom is entangled in the parents unconscious and the mythology of that family, there are many layers of truth and of enjoyment. Yet, Bonneuil's focus is on giving place to the child's struggle with his symptom in order to free and articulate his desire. Giving this place to the symptom may mean a crossing into madness, or at least proximity with the madness in all of us, as well as the child's own deep suffering. This may be why this more dense psychic space is punctuated by travels, apprenticeships, public performances. We all find some way to intercalate that which is so intimate and near madness to the identities and semblants that are the public offerings, and we are recognized for such efforts. This is not adjustment but rather making a place for those “troubled” children in the social, each art show or performance, the child's work, future profession or trade, serve as part of the opening of desire, rendering the outside space as a place where one is no longer a psychiatric case, but a person's making his or her way through life. So that the space between places, between the structural and literal inside that marks the institution and its openings to the outside, foster a subjective oscillation between, as Vanier notes, “flight” and “project.” Led by the ethics of one's desire, the desire of the educator (Vanier, 2004), of the adults (Moraes, 1995a), one could expect that Bonneuil will never be a model for other institutes, but rather, a place of interrogation.

It would seem that increased sensitivity to the political alliances and misalliances of working in “mental health” does not dis-qualify such critical projects embracing places where madness is respected and its suffering addressed in favor of the subject. The recent abundance of auto-biographies of folks with autism, borderline diagnosis, their forms of suffering and who are troubled (to use Vanier's words) by the current systems may ask for a new response from us, within an appreciation of their struggle for identity and their desire to feel less marginalized. We may listen and recognize their other voices in various public media. Such responses could well include psychanalytic frames—itself a frame of listening, of giving place to subjectivity—if psychoanalysis moves away from serving a cohort of “suitable” patients and toward the creativity that lies within its particular embrace of the question of human desire (Moraes, 1995b).

## Ethics statement

The paper is exempt from ethics review as it represents the view of design and principles that guide a day hospital that is accredited by the state. No particular human subject is discussed and at maximum it could be seen as ethnographic study of a public domain. The paper is written by the Director and there appears to be no ethical issues regarding a historical review of the principles of a children's day hospital. Many of the procedures described are already recorded in a documentary film cited in references.

## Author contributions

The description of the operation of The Experimental School in Bonneuil-sur-Marne was written by CV its director. CV obviously knows the process of Bonneuil given her position. CV's essay was somewhat edited to change it to a form more amenable to a written piece. The Commentary was written by KM. who added relevant English references. Both were checked over by CV.

### Conflict of interest statement

The authors declare that the research was conducted in the absence of any commercial or financial relationships that could be construed as a potential conflict of interest.

## Footnotes

^1^Many of these categories have changed nomenclature since the DSM-5 and its coordination with the ICD-10. Mental Retardation is now “Intellectual Disability,” we now diagnose someone within the autism spectrum, and the psychoses are parsed according to their phenomenological appearance and neurology, the latter is a particularly important dimension of the DSM-5 (American Psychiatric Association, 2013).

^2^Please see commentary for further exposition on these debates.

^3^France's clinical practitioners do not necessarily wholly subscribe to the diagnostic categories of the DSM-5, despite pressure from parents groups/governmental agencies. There is increasing parental pressure to emulate the North American model, itself now becoming complicated by organizations, and patient and austism self-advocacy groups who see the hegemony of the neuorotypical as a neuro-biological oppression of difference (Brownlow and O'Dell, 2013). John Sinclair‘s call for a positive autistic identity, conferences that are organized to respect the needs of autistic persons and recognize their self-understanding and narratives have also expressed hostility to psychoanalysis as well as opposition to neurotypical insensitivity to the autistic perspective. Their efforts do suggest the place of the social link in addressing this wide collection of people. The ideology of North American self-advocacy groups have not been as influential in France where groups, such as Satedi, are more focused on education and addressing the difficulties of such individuals (Chamak and Bonniau, 2013). Identity politics are viewed differently, even with the new neuro-biological category from which to claim rights and social recognition. Such movements deserve further discussion, as they have affected the diagnosis of psychosis and refocused from an address to the subject who lives with some form of unassimilable behavior and social difficulty to re-training through CBT (Laurent, 2012; O'Loughlin and Merchant, 2012).
